# EGF Up-Regulates *miR-31* through the C/EBPβ Signal Cascade in Oral Carcinoma

**DOI:** 10.1371/journal.pone.0108049

**Published:** 2014-09-17

**Authors:** Wen-Cheng Lu, Shou-Yen Kao, Cheng-Chieh Yang, Hsi-Feng Tu, Cheng-Hsien Wu, Kuo-Wei Chang, Shu-Chun Lin

**Affiliations:** 1 Institute of Oral Biology, National Yang-Ming University, Taipei, Taiwan; 2 Department of Dentistry, National Yang-Ming University, Taipei, Taiwan; 3 Department of Dentistry, National Yang-Ming University Hospital, Yi-Lan, Taiwan; 4 Department of Stomatology, Taipei Veterans General Hospital, Taipei, Taiwan; IPMC, CNRS UMR 7275 UNS, France

## Abstract

Oral squamous cell carcinoma (OSCC) is one of the most prevalent carcinomas worldwide. MicroRNAs (miRNAs) are short, non-coding RNAs that regulate gene expression and modulate physiological or pathological processes including OSCC carcinogenesis. *miR-31* has been found to be up-regulated in OSCC and to act as an oncogenic miRNA. However, the molecular mechanism underlying *miR-31* up-regulation in OSCC is still obscure. The activation of epidermal growth factor receptor (EGFR) signaling axis plays key roles in driving oral carcinogenesis. Our screening identified that there is up-regulation of *miR-31*, *miR-181b* and *miR-222* in OSCC cells following EGF treatment. Subsequent analysis showed that EGF treatment led to AKT activation, which then resulted in *miR-31* up-regulation. Moreover, EGF treatment and the AKT activation induced by exogenous expression up-regulated C/EBPβ expression. The *miR-31* up-regulation induced by EGF was abrogated by AKT inhibition or by the knockdown of C/EBPβ expression. In OSCC cell subclones stably overexpressing the functional isoform of C/EBPβ, *miR-31* expression was up-regulated. Curcumin is a natural ingredient exhibiting anti-cancer potential. It was found that curcumin attenuated AKT activation and the up-regulation of C/EBPβ and *miR-31* caused by EGF stimulation in OSCC cells. Lastly, concordance across the expression of EGFR, the expression of C/EBPβ and the expression of *miR-31* in OSCC tissues was found. This study describes a novel scenario where the up-regulation of *miR-31* expression in OSCC is, at least in part, a consequence of EGFR oncogenic activation. Although the AKT activation and C/EBPβ expression after EGF treatment might not be directly linked, both events are the crucial mediators underlying *miR-31* up-regulation in the EGFR signaling axis.

## Introduction

Head and neck carcinoma, including oral squamous cell carcinoma (OSCC), is the fifth most common cancer worldwide [1]–[4]. Epidermal growth factor receptor (EGFR) encodes a transmembrane protein that can be activated by either epidermal growth factor (EGF) or transforming growth factor α (TGFα); such activation promotes oncogenesis [5]. EGFR activation triggers various intracellular signaling networks such as the activation of extracellular signal-regulated kinases (ERKs), which are related to the mitogen-activated protein kinases (MAPKs), to AKT (protein kinase B) and to other similar kinases [3], [5]. Amplification and/or overexpression of EGFR is prevalent in OSCC and the activation of EGFR downstream elements seems to play a key role in driving OSCC pathogenesis [6]–[11].

MicroRNAs (miRNAs) are non-coding double-stranded RNAs that consist of approximately 22 nucleotides. miRNAs bind to complementary sites in the 3′untranslated regions of their targeted gene; this causes either translational inhibition or degradation of the targeted mRNA [12]. Aberrant expression of *miR-21*, *miR-24*, *miR-31*, *miR-134*, *miR-181a/*-*181b*, *miR-184*, *miR-196a/*-*196b*, *miR-211*, *miR-221/*-*222* and other miRNAs is known to play an important role in the development and progression of OSCC [2], [4], [12]–[22]. Our previous study identified that *miR-31* is enhanced among malignant phenotypes and when there is OSCC tumorigenesis [2]. In addition, *miR-31* has been shown to activate hypoxia pathways through targeting of the *FIH* gene [2]. *miR-31* has also been found to be associated with oncogenesis in other malignancies [23]–[25]. In addition, up-regulation of *miR-31* has been found in both OSCC tissue samples and the plasma of patients [2], [14]. One of our recent studies identified the up-regulation of *miR-31* in oral premalignant disorders. *miR-31* also plays a role in the immortalization of normal oral keratinocytes (NOK) [1]. Another recent study depicted that *miR-31* is transcribed from sequences within the first intron of the non-coding RNA LOC554202 [26]. It has been suggested that the transcription level of *miR-31* parallels the expression level of LOC554202. Hypermethylation in the CpG islands of the promoter region of this gene silences the expression of both LOC554202 and *miR-31* [27]. Oncogenic signals modulating promoter activity of LOC554202 have been postulated to mediate changes in *miR-31* expression during the neoplastic process [28].

The basic leucine zipper transcription factor CCAAT/enhancer binding protein (C/EBP) family contains six members (α–ζ). These proteins are members of the basic leucine zipper transcription factor group and are important mediators of various physiological and pathological states including tumorigenesis [29]. C/EBPα plays a suppressor role in OSCC and other keratinocytic malignancies by maintaining cellular homeostasis [30], [31]. Various lines of evidence indicate that C/EBPβ is an oncogenic factor. *C/EBPβ* gene maps to human chromosome 20q13, a hot spot region frequently amplified in OSCC [32]. The gene encodes several N-terminally truncated protein isoforms. Isoform 2 (encoded by *C/EBPβ-2* transcript*)* is a transcriptional activator that modulates pathogenesis in many systems; however the product of *C/EBPβ-1* also acts to antagonize C/EBPβ-2 activity as part of a balance mechanism [33]. C/EBPβ plays very important roles in the pathogenesis of keratinocytes. Specifically, the protein modulates the growth and differentiation of keratinocytes [34] as well as cooperating with Ras and being able to suppress p53 during the transformation of keratinocytes [34]–[36]. Nonetheless, the oncogenic stimuli and the activated signaling cascades that are able to up-regulate C/EBPβ during OSCC have not been addressed up to the present.

Curcumin is a polyphenol derived from *Curcuma longa* and is abundant in the Indian spice turmeric, which is a common food ingredient throughout the world [37]. It mediates pluripotency by inhibiting various oncogenic signaling pathways including AKT, ß-catenin, Bcl2, ERK, NFκB and others [37], some of which seem to be involved in counteracting EGFR stimulation. Furthermore, curcumin activates p38/MAPK bringing about C/EBPα up-regulation in oral keratinocytes, which results in tumor suppression [31]. In this study, we identified for the first time that EGF is an oncogenic factor that is able to up-regulate *miR-31* expression in OSCC cells. C/EBPβ was found to be an essential effector of this up-regulation. Furthermore, the EGFR-AKT-C/EBPβ-*miR-31* regulatory axis in OSCC cells was found to be attenuated by curcumin.

## Materials and Methods

### Cell culture and reagents

The OSCC cell lines HSC-3, OECM-1 and SAS, NOK primary culture cells and Phoenix package cells were cultivated as previously described [1], [2]. Curcumin, EGF, dimethyl sulfoxide (DMSO), LY294002 and U0126 were purchased from Sigma-Aldrich (St. Louis, MO). Curcumin, LY294002 and U0126 were dissolved in 0.5% DMSO as working solutions for treatment. si-C/EBPβ and a scramble (si-Scr) control oligonucleotide were purchased from Santa Cruz Biotech (Santa Cruz, CA). TransFectin Lipid Reagent (Bio-Rad, Hercules, CA) was used as the transfection reagent. Unless specified, all other reagents were purchased from Sigma-Aldrich.

### qRT-PCR analysis

The expression of a panel of miRNAs was analyzed using the TaqMan MicroRNA Assay system according to the manufacturer’s instructions (Applied Biosystems; Foster City, CA) using *U6B* small nuclear RNA as the control. The mRNA expression levels of *C/EBPβ* gene and *FIH* gene were analyzed by the TaqMan qPCR Assay system (Applied Biosystems) using *GAPDH* as the internal control. The threshold cycle (Ct) method was used to measure the relative changes in expression of the various RNAs. ΔΔCt is the difference in ΔCt values between the sample groups and the experimental settings. The 2^−ΔΔCt^ represents the fold of change in expression [2].

### Western blot analysis

Cell lysate was isolated, separated by SDS-PAGE and transferred to a nitrocellulose membrane. The membrane was then incubated with appropriate primary antibodies (Table 1) using a previously described protocol [2]. This was followed by incubation with anti-mouse or anti-rabbit secondary antibodies (Chemicon Int. Inc., Billerica, MA) as appropriate at 1:1000 dilution. Signals were detected by a Western Lightening Chemiluminescence Reagent Plus kit (Perkin-Elmer, Wellesley, MA) and a Fusion SL imaging system measurement system (Viber Lourmat, Marne-La-Valee, France). The expression levels of the proteins of interest were normalized against the expression level of GAPDH.

**Table 1:** Primary antibodies used in the present study.

Antibody	MW (kDa)	Host	Dilution	Supplier	Cat. No.
Phosphorylated (p)-AKT	62	Mouse	1∶1000	Cell Signaling	4051
Total (t)-AKT	62	Mouse	1∶1000	Santa Cruz Biotech	SC-5298
β-catenin	92	Mouse	1∶1000	BD Biosciences	610154
Bcl2	26	Mouse	1∶1000	Santa Cruz Biotech	SC-7382
C/EBPβ	36	Rabbit	1∶1000	Abcam	Ab137510
C/EBPβ* ^,^ +	36	Mouse	1∶50* or 1∶200+	Santa Cruz Biotech	SC-7962
EGFR*	175	Rabbit	1∶50	Novo Castra	NCL-EGFR-384
Phosphorylated (p)-EGFR+	175	Rabbit	1∶200	Cell Signaling	2234
Phosphorylated (p)-ERK	42/44	Rabbit	1∶1000	Cell Signaling	9101
Total (t)-ERK	42/44	Rabbit	1∶1000	Cell Signaling	9102
GAPDH	36	Mouse	1∶10000	Santa Cruz Biotech	SC-32233

*IHC analysis;

+ IF analysis.

### Plasmids

pUSE-AKT and the vector alone (VA) control plasmid, which were used for transient expression, were provided by Dr. Yang, Cheng-Chieh. For exogenous expression of functional C/EBPβ, the *C/EBPβ-2* cDNA sequence was cloned into the pBabe-puro vector in order to produce retroviruses for infection [2]. Stable C/EBPβ cell subclones were established by puromycin selection. VA cell subclones were used as the control cells after they had been infected with the control virus.

### Tissue microarray (TMA)

*The fabrication of the tissue microarray* (TMA), which consisted of sixty OSCC tissue cores and some paired non-cancerous oral mucosa (NCOM) cores (Table 2), was approved by The Institutional Review Board of National Yang-Ming University Hospital. This OSCC TMA was constructed using a previously published method [7].

**Table 2:** Clinicopathological parameters of OSCC.

n =	60
Age (Mean ± SE years)	54.7±1.6
Gender (Male/Female)	52/8
TNM staging	
T1–3	17
T4	43
N0	42
N+	18
Stage I	4
Stage II	7
Stage III	6
Stage IV	43

### Immunohistochemistry (IHC)

The tissue sections of TMA were de-paraffinized and were subjected to an antigen retrieval process in a 2100 Antigen Retriever Autoclave device (Prestige Medical, Northridge, CA). EGFR and C/EBPβ immunoreactivity levels were detected by immunohistochemistry using previously described protocols [2]. Incubation with primary antibodies (Table 1) was carried out at 4°C overnight. Preimmunized mouse IgG was used as a negative control. The immunoreactivities were captured by Image-Pro software (Media Cybernetic, Rockville, MD) and quantified by pixel analysis using Photoshop software (Adobe; San Jose, CA) according to protocols that have been previously described [1], [38].

### *In situ* hybridization (ISH)

Digoxigenin-labeled *miR-31* probe, control scramble probe and the reagents required for ISH were purchased from Exiqon (Vedbaek, Denmark) [1], [39]. Sections of TMA were fixed, then incubated in pre-hybridization buffer; this was followed by hybridization with 10 µM each of *miR-31* probe and scramble probe overnight. Slides were then washed and incubated with anti-digoxigenin antibody. NBT/BCIP reagent was then used to detect the ISH signals. The *miR-31* staining was captured and quantified using the same methods as were used for IHC [1].

### Immunofluorescence (IF)

After antigen retrieving, tissue sections were incubated with appropriate primary antibodies (Table 1) at 4°C overnight. This was followed by incubation in fluorescent-conjugated secondary antibodies (Alexafluor-488 and Alexafluor-594; Jackson Immunoresearch Lab, Suffolk, UK) at 1:200 dilution. The nuclei were also stained with DAPI. Images were captured using a FV1000 Confocal Microscope (Olympus. Tokyo, Japan).

### Statistical analysis

The Graphic algorithm was used for the cluster analysis [2]. Unpaired *t*-tests and linear regression analysis were used for statistical analysis. A *p* value of <0.05 was considered statistically significant.

## Results

### EGF up-regulates miR-31 expression via the AKT signaling pathway

**Figure 1:**
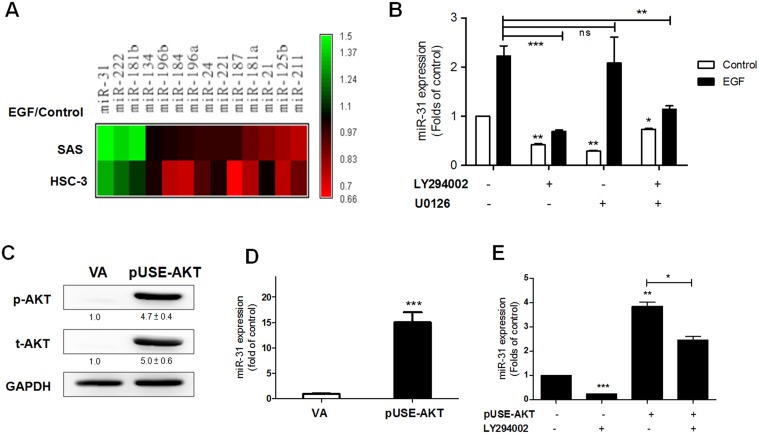
EGF up-regulates *miR-31* expression by virtue of AKT activation in OSCC cells. (A) The Graphic algorithm was used to illustrate the expression profile of 14 miRNAs following EGF treatment in SAS and HSC-3 cells. Green, up-regulated; red, down-regulated. (B–E) SAS cells. (B, D, E) qRT-PCR analysis. (C) Western blot analysis. (B) Inhibition of potential EGF downstream signals by pretreatment with LY294002 and U0126. LY294002 decreased endogenous *miR-31* expression as well as EGF induced *miR-31* expression. (C) Exogenous AKT expression and AKT activation mediated by plasmid transfection. (D) Up-regulation of *miR-31* expression induced by AKT activation. (E) LY294002 administration blocked both endogenous *miR-31* expression and AKT induced *miR-31* expression. VA, vector alone. Numbers below pictures are normalized values. Data in (A) are from duplicate experiments. Other data are the means ± SE from at least triplicate analysis. *ns*, not significant; *, *p*<0.05; **, *p*<0.01; ***, *p*<0.001; un-paired *t*-test.

The expression of 14 oncogenic miRNAs following treatment with 100 ng/mL EGF for 24 hours was analyzed using SAS and HSC-3 cells [2], [4], [12]–[22]. Algorithm analysis was used to create a profile of miRNA expression as modulated by EGF (Fig. 1A). *miR-31*, *miR-181b* and *miR-222* were up-regulated by EGF to different degrees in both cell lines. The up-regulation of *miR-31* was the highest among these three miRNAs. EGF had no influence, produced inconsistent changes in expression level or gave rise to a slight down-regulation in expression when the remaining miRNAs were examined. To clarify the signaling pathway involved in EGF induced *miR-31* expression, SAS cells were pretreated with 10 µM LY294002 to block AKT activity and 10 µM U0126 to block ERK activity for 2 hours, and then *miR-31* expression was analyzed following EGF stimulation. Both LY294002 and U0126 treatment suppressed endogenous *miR-31* expression, while only LY294002 inhibited EGF induced *miR-31* up-regulation (Fig. 1B). Combined inhibition with both LY294002 and U0126 also decreased both endogenous *miR-31* expression and the EGF induced *miR-31* expression. SAS cells were then transfected with the pUSE-AKT plasmid for 24 hours. Western blot analysis showed increased expression of both total AKT and phosphorylated (p)-AKT after transfection (Fig. 1C); this treatment also resulted in an increase of *miR-31* up-regulation (Fig. 1D). The up-regulation of *miR-31* expression induced by exogenous AKT expression was attenuated when the cells were treated with LY294002 (Fig. 1E). These findings suggest that EGF up-regulates *miR-31* expression via the AKT signaling pathway.

### EGF-AKT signaling cascade induces C/EBPβ expression

**Figure 2:**
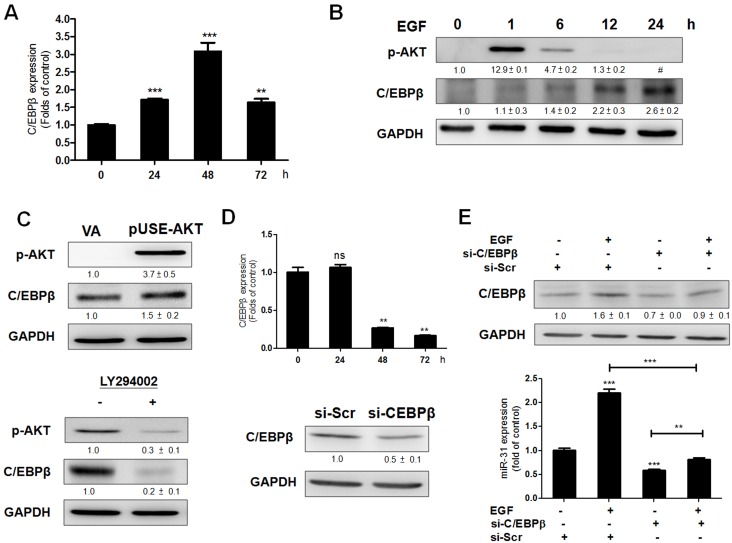
EGF induces C/EBPβ expression in SAS cells. (A; D, Upper; E, Lower) qRT-PCR analysis. Others, Western blot analysis. (A) EGF treatment up-regulated *C/EBPβ* mRNA expression at 24 h, 48 h and 72 h. (B) After EGF treatment, AKT activation peaked as early as 1 h and then decreased at 6 h to reach its basal level after 12 h, while C/EBPβ increased at 6 h and lasted after 24 h. (C) Activation of AKT by plasmid transfection for 24 h (Upper) and treatment with LY294002 for 24 h (Lower) increased and decreased C/EBPß expression, respectively. (D) Transfection with 100 nM si-C/EBPβ oligonucleotide down-regulated *C/EBPβ* mRNA expression at 48 h and 72 h (Upper); it also down-regulated C/EBPβ protein expression at 48 h (Lower). (E) EGF treatment for 48 h up-regulated C/EBPβ protein expression (Upper) and *miR-31* expression (Lower). This up-regulation was attenuated after transfecting with si-C/EBPβ oligonucleotide. Numbers below Western blot pictures are normalized values. #, quantitation unavailable due to faint image signals. Data are the means ± SE from at least triplicate analysis. *ns*, not significant; **, *p*<0.01, ***, *p*<0.001; un-paired *t*-test.

Since C/EBPβ is able to transactivate the LOC554202 locus [28], we wondered if C/EBPβ is a downstream effector of the EGFR-AKT signaling cascade in OSCC cells. To this end, SAS cells were treated with EGF for different time periods. The analysis indicated that *C/EBPβ* mRNA expression was up-regulated by EGF (Fig. 2A). Western blot analysis further revealed that the activation of AKT occurred early, within 6 hours after treatment, while C/EBPβ expression increased progressively after AKT activation had begun (Fig. 2B). Moreover, C/EBPβ expression increased after transfecting with pUSE-AKT plasmid (Fig. 2C, Upper). In contrast, treatment with LY294002, which markedly abrogated endogenous AKT activity, resulted in an obvious decrease in the expression level of C/EBPβ (Fig. 2C, Lower). Therefore, it would seem that the EGFR-AKT signaling cascade is able to modulate C/EBPβ expression.

### The EGF- C/EBPβ cascade up-regulates miR-31 expression

Knockdown of C/EBPβ expression using si-C/EBPβ results in down-regulation of *C/EBPβ* mRNA expression (Fig. 2D, Upper). Endogenous C/EBPβ protein expression is also knocked down by si-C/EBPβ (Fig. 2D, Lower). C/EBPβ up-regulation (Fig. 2E, Upper) and *miR-31* up-regulation (Fig. 2E, Lower), which are both induced by treatment of EGF, are also attenuated by this knockdown.

**Figure 3:**
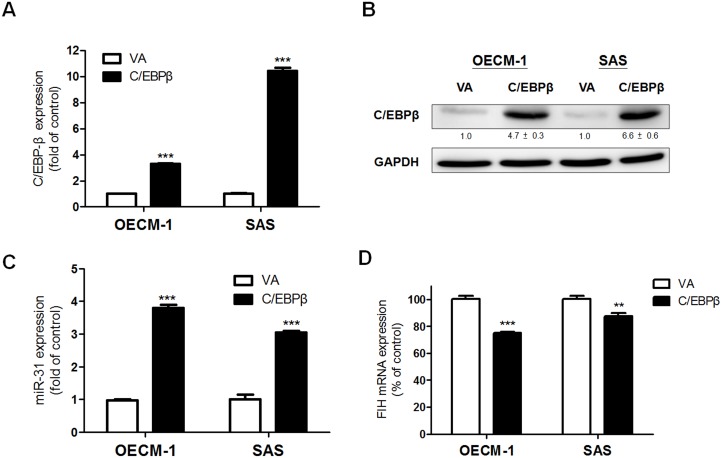
OSCC cell subclones overexpressing C/EBPβ show *miR-31* up-regulation. (A, C, D) qRT-PCR analysis. (B) Western blot analysis. Stable OECM-1 and SAS cell subclones exhibit (A) exogenous *C/EBPβ* mRNA expression, and (B) protein expression. Exogenous C/EBPβ protein expression results in significant (C) up-regulation of *miR-31* and (D) down-regulation of *FIH* mRNA expression. The numbers below the pictures are normalized values. Data are the means ± SE from at least triplicate analysis. **, *p*<0.01, ***, *p*<0.001; un-paired *t*-test.

During our preliminary experiments we found that C/EBPβ-2 protein expression was present in OSCC cells, while C/EBPβ-1 protein expression was absent in OSCC cells (data not shown). Based on these findings, *C/EBPβ-2* cDNA was cloned into pBabe retroviral vector to allow delivery. After infection with a retrovirus carrying the *C/EBPβ-2* coding sequence, stable OECM-1 and SAS cell subclones were established by puromycin selection. These stable cells exhibited exogenous *C/EBPβ* mRNA and protein expression (Fig. 3A and Fig. 3B, respectively). Consistent with our pervious study, where *miR-31* was found to suppress *FIH* expression in OSCC cells [2]. The up-regulation of *miR-31*, which had earlier been found to be secondary to C/EBPβ expression (Fig. 3C) together with down-regulation of *FIH* mRNA expression (Fig. 3D), were also seen in these stable cells. Thus, EGF induced C/EBPβ up-regulation is able to affect *miR-31* expression in OSCC cells.

### Curcumin down-regulates miR-31 expression in oral keratinocytes

**Figure 4:**
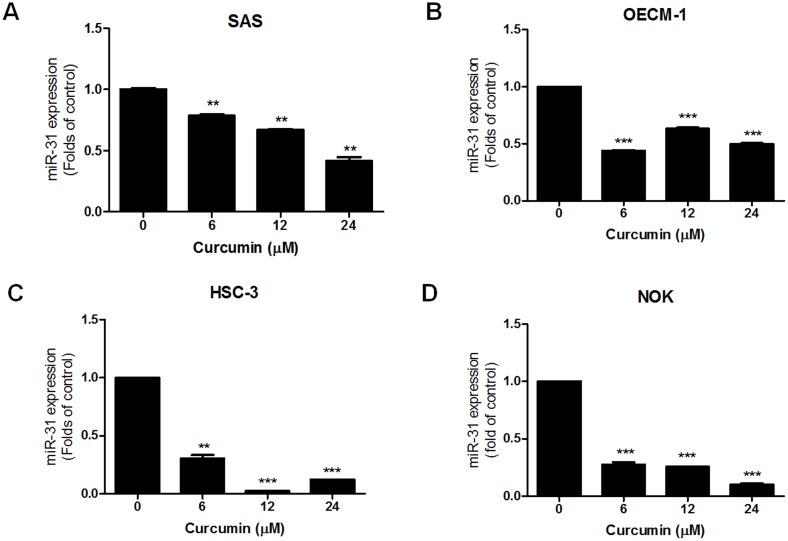
Down-regulation of *miR-31* expression following treatment with curcumin in oral keratinocytes. qRT-PCR analysis. (A) SAS cell. (B) OECM-1 cells. (C) HSC-3 cells. (D) NOK primary culture cells. Data are the means ± SE from triplicate analysis. **, *p*<0.01; ***, *p*<0.001; un-paired *t*-test.

Curcumin was known to suppress oncogenicity and therefore we tested the effect of curcumin on the abrogation of *miR-31* expression in OSCC cells. SAS, OECM-1 and HSC-3 cells were treated with serially diluted curcumin (0, 6, 12 and 24 µM) for 24 hours, and expression of *miR-31* was analyzed. *miR-31* expression was found to be down-regulated in a dose-dependent manner in SAS cells (Fig. 4A). The down-regulation of endogenous *miR-31* expression following curcumin treatment also occurred in the OECM-1 cells (Fig. 4B) and HSC-3 cells (Fig. 4C). Following curcumin treatment, *miR-31* expression in NOK cells was also found to be down-regulated in a dose-dependent manner (Fig. 4D).

### Curcumin attenuates miR-31 expression via AKT inhibition in OSCC cells

**Figure 5:**
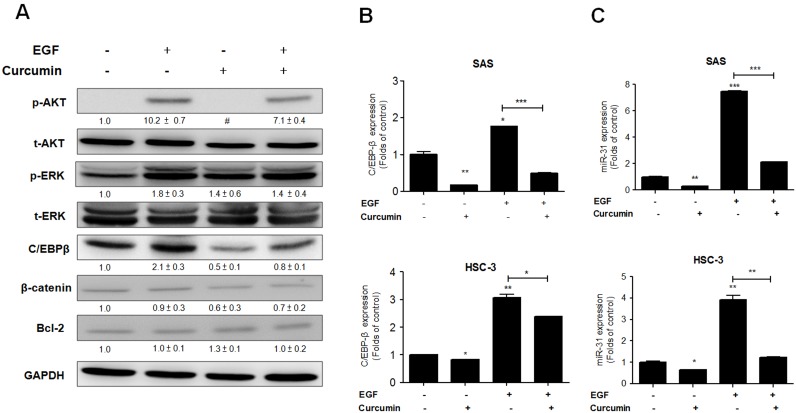
Curcumin down-regulates *miR-31* expression via EGF downstream signals in OSCC cells. (A) Western blot analysis. (B, C) qRT-PCR analysis. (A) SAS cells. (B, C) SAS cells (Upper) and HSC-3 cells (Lower). (A) The analysis shows the attenuation of EGF induced AKT activation and C/EBPβ up-regulation after treatment with 12 µM curcumin for 24 h. EGF induced ERK activation was not obviously attenuated by curcumin. Treatment with EGF or curcumin had little effect on the expression of β-catenin or Bcl2. (B) Curcumin treatment down-regulates endogenous *C/EBPβ* mRNA expression and EGF induced *C/EBPβ* mRNA expression in SAS cells and HSC-3 cells. (C) Curcumin attenuates endogenous *miR-31* expression and EGF induced *miR-31* expression in both types of cell. The numbers below the pictures are normalized values. #, quantification unavailable due to faint image signals. Data are the means ± SE from at least triplicate analysis. *, *p*<0.05; **, *p*<0.01; ***, *p*<0.001; un-paired *t*-test.

We further explored the effect of curcumin on EGF downstream signaling in SAS cells. Western blot analysis showed that there was activation of AKT and ERK following EGF stimulation, and that this AKT activation was able to be attenuated by 12 µM curcumin (Fig. 5A). However, in contrast to the above, curcumin did not bring about a consistent modulation of ERK signaling. The assays also revealed that ß-catenin expression was slightly down-regulated by curcumin, whereas the expression levels of Bcl-2 were not affected by curcumin. Interestingly, the level of C/EBPβ expression seemed to parallel the AKT activation status as modulated by either EGF or curcumin. In addition, the effect of EGF on *C/EBPβ* mRNA expression was also attenuated by curcumin in both SAS cells (Fig. 5B, Upper) and HSC-3 cells (Fig. 5B, Lower). In addition, the *miR-31* expression level, whether elicited by EGF or not, was also attenuated by curcumin in both SAS cells (Fig. 5C, Upper) and HSC-3 cells (Fig. 5C, Lower). These findings suggest that curcumin is able to attenuate EGF induced *miR-31* expression via the blocking of AKT signaling and, furthermore, C/EBPβ seems to be involved in this regulation.

### Higher levels of EGFR, C/EBPβ and miR-31 expression in OSCC tumor tissues than in non-cancerous oral mucosa

**Figure 6:**
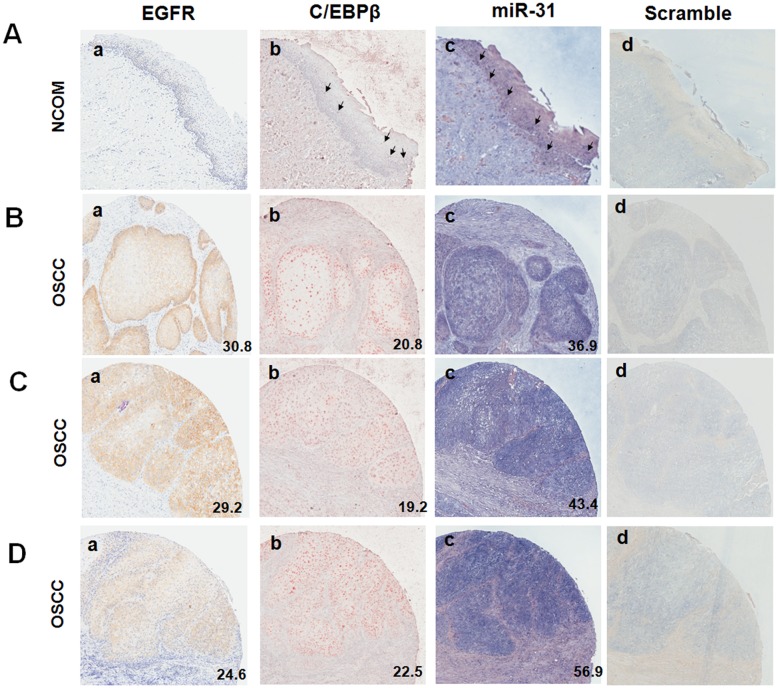
Immunoreactivity of EGFR and C/EBPβ, together with staining of *miR-31* in representative TMA tissues. (A), a NCOM tissue. (B–D), three individual OSCC tissues. a to d were consecutive TMA sections from the same sample. (a, b) Immunohistochemistry of EGFR and C/EBPβ, respectively. (c, d) *In situ* hybridization of *miR-31* probe and scramble probe, respectively. Cytosolic and/or membranous brown-red EGFR immunoreactivity, nuclear and cytosolic brown-red C/EBPβ immunoreactivity, and cytosolic and nuclear bluish *miR-31* are considered positive signals. Arrows in (A) indicate the representative positive signals in NCOM. The digital scores for the tumor samples were obtained by pixel analysis quantification and are shown in right lower corner of each picture. 100x magnification.

In order to validate the presence of the EGFR-C/EBPβ-*miR-31* regulatory axis in OSCC tissues, IHC and ISH were performed on a TMA containing tumor tissues and NCOM tissues (Table 2). There were scanty membranous EGFR and scattered nuclear C/EBPβ immunoreactivities in the NCOMs available for analysis (Fig. 6Aa, b). Sporadic *miR-31* staining in basal or parabasal cells was present in the epithelial layer of NCOMs (Fig. 6A, c), which is similar to the results that we previously detected in normal oral epithelium [1]. Various cytosolic and membranous EGFR immuoreactivities (Fig. 6B–D, a), together with nuclear and faint cytosolic C/EBPβ immuoreactivities (Fig. 6B–D, b), and cytosolic and nuclear *miR-31* staining (Fig. 6B–D, c), were seen in the tumor tissues. In contrast, scramble probe gave barely detectable background staining of cells in consecutive tissue sections. (Fig. 6A–D, d). The brown-red pixels of EGFR and C/EBPβ; and the blue pixels of *miR-31* in consecutive OSCC tissue sections were calculated. The EGFR signals were scored over the range 4.6–56.2, the C/EBPβ signals were scored over the range 17.2–44.2, and the *miR-31* signals were scored over the range 12.0–68.4, in the various tumor tissues. The OSCC tumor tissue samples had stronger EGFR, C/EBPβ and *miR-31* expression than the NCOM samples.

### Correlation between EGFR, C/EBPβ and miR-31 expression in OSCC tissues

**Figure 7:**
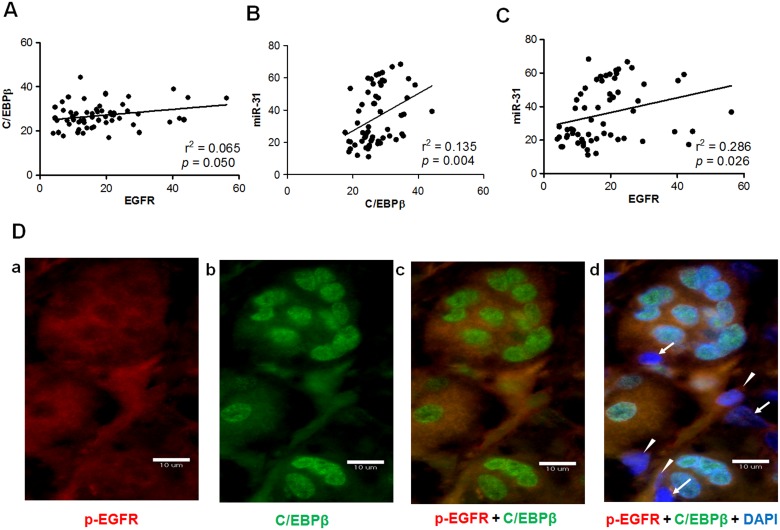
Linear regression analysis of pixel scores of EGFR, C/EBPβ, and *miR-31*, and immunofluorescence analysis. (A–C), Correlation between the scores of C/EBPβ and EGFR, between the scores of C/EBPβ and *miR-31*, and between the scores of EGFR and *miR-31*, respectively. Significant correlations were found between the scores of each pair of molecules. (D) Immunofluorescence of a representative OSCC tumor. a, phosphorylated (p)-EGFR, red fluorescence; b, C/EBPβ, green fluorescence. c, overlapping of p-EGFR and C/EBPβ. d, overlapping of picture c and the staining of DAPI. The images reveal the presence of p-EGFR and nuclear C/EBPβ in the vast majority of tumor cells. In addition, the presence of tumor cells exhibiting the absence of both p-EGFR and nuclear C/EBPβ expression are indicated by arrows. Furthermore, the presence of tumor cells exhibiting p-EGFR expression when C/EBPβ expression is absent are indicated by arrow heads. Bars, 10 µm.

Linear regression analysis of the pixel scores showed that, in OSCC tissues, there was a significant correlation between the expression of EGFR and the expression of C/EBPβ (Fig. 7A), between the expression of C/EBPβ and the expression of *miR-31* (Fig. 7B), and between the expression of EGFR and the expression of *miR-31* (Fig. 7C). The correlation between C/EBPβ and *miR-31* was rather strong, while the correlation between EGFR and C/EBPβ was relatively weaker. This poses the question as to whether EGFR activation and downstream activation of C/EBPβ co-exist in tumor cells. IF was performed to detect phosphorylated (p)-EGFR and nuclear C/EBPβ in tumor tissues. Co-existence of p-EGFR signal (Fig. 7D, a) and nuclear C/EBPβ signal (Fig. 7D, b) can be found in a large fraction of the tumor cells which were analyzed, which implies the parallel presence of EGFR and C/EBPβ co-activation in these tumor cells (Fig. 7D, c, d). Since the inactivation of both EGFR and C/EBPβ can also be seen in tumor cells (Fig. 7D, d; arrows), this further supports the hypothesis that there is a concordance between the activation of EGFR and the activation of C/EBPβ. Interestingly, there were some tumor cells that exhibited EGFR activation but no C/EBPβ activation (Fig. 7D, d; arrow heads). Thus it is possible that C/EBPβ is not activated in some tumor cells despite there having been EGFR activation.

## Discussion

EGFR overexpression has been shown to be associated with a worse OSCC prognosis [10], [11]. Upon binding with ligands, activated EGFR regulates cell growth, differentiation, motility and tumorigenesis; EGFR does this by triggering multiple signaling pathways [5]. Our previous studies have defined a number of crucial roles for *miR-31* in determining the oncogenic behavior of OSCC and *miR-31* expression could be an important marker for early diagnosis of OSCC [1], [2], [14], [15]. Many mechanisms, including genomic alterations, aberrances in epigenetic regulation, defects in the transcription and processing machineries and others, are thought to underlie the disruption of miRNA expression in tumors [4]. This study highlights a new set of clues demonstrating that EGF administration (EGFR activation) is able to up-regulate *miR-31* expression. Using an AKT blocker and an ERK blocker, our approaches show that AKT signaling is the key mediator of this up-regulation in OSCC cells. The induction was only slight and the most likely reason for this is the presence of both high EGFR activity and high endogenous *miR-31* expression in the tumor cells [2], [9]. Nevertheless, the blocking of the EGFR-AKT cascade resulted in a rather conspicuous reduction in the level of expression of *miR-31* in OSCC cells.

Previously, Seike *et al* [40] has identified that activation of EGFR up-regulates *miR-21* in lung carcinoma cells. Our findings define a novel carcinogenic role for EGFR, namely the up-regulation of another oncogenic miRNA that is crucial to OSCC pathogenesis. Apart from OSCC, *miR-31* has also been found to be oncogenic in lung, cervical and colorectal carcinomas [23]–[25]. Therefore, the induction of *miR-31* by EGF stimulation might also be crucial to the development of other types of malignancies. In this context, the modulation effect of EGFR on the expression of *miR-181b* and *miR-222* in OSCC and other malignancies needs to be further elucidated [20], [21].

C/EBPβ plays important roles in the differentiation and transformation of keratinocytes by driving complicated regulation [34]–[36]. Oncogenic Ras has been reported to induce C/EBPβ transactivation in keratinocytes [36]. This study presents new evidence highlighting the fact that EGFR-AKT signaling is able to up-regulate C/EBPβ expression by acting as an upstream stimulator. In addition, the findings from both knockdown and overexpression in this study substantiate the modulation effect of C/EBPβ on *miR-31* expression during OSCC pathogenesis. However, since AKT is an early event in the signaling cascade controlling C/EBPβ induction by EGF, and these two events are not directly linked, other still unidentified molecular mediators are probably intervening.

This study identified faint EGFR immunoreactivity, scattered C/EBPβ immunoreactivity and sporadic *miR-31* staining in non-cancerous oral mucosa, which is in agreement with our previous studies as well as those of others [1], [9], [33]. Since EGFR and *miR-31* are highly expressed in OSCC tissues [2], [9], [10], in this study we were able to further clarify that there is concordance in the expression of EGFR, C/EBPβ and *miR-31* in OSCC samples. C/EBPβ is crucial for the homeostasis and pathogenesis of keratinocytes [34]–[36]. It is also well known that when respiratory epithelium cells are exposed to tobacco condensate, there is induction of C/EBPβ expression and that this in turn transactivates the *miR-31* host locus [28]. It is thus very likely that C/EBPβ up-regulation may also underlie the up-regulation of *miR-31* expression in cervical and lung carcinomas [24], [25]. We also found that when EGFR is activated, some tumor cells do not show C/EBPβ activation. This paradox might occur for a number of reasons including the presence of an impairment that affects the AKT-C/EBPβ cascade, the fact that there is alternative modulation taking place, or the presence of a confounding effect involving an anatogonist C/EBPβ isoform that is present in a tumor cell subpopulation.

Curcumin modulates signaling cascades and suppresses OSCC carcinogenesis [31]. This study found specifically that AKT is the main EGFR downstream signal that is suppressed by curcumin in SAS cells. Curcumin has also been reported to inhibit the proliferation of cisplatin-resistant ovarian cancer cells through AKT inactivation [41]. Furthermore, curcumin has been reported to up-regulate *miR-22* expression and down-regulate miR-199a* expression in pancreatic cancer cells [42]. The findings of the present study demonstrate that curcumin is able to down-regulate *miR-31* expression in OSCC cells by inhibiting AKT rather than ERK. In addition, our preliminary results suggest that curcumin is able to slightly repress ß-catenin expression in SAS cells [37]. It would be interesting to further elucidate whether curcumin may affect the activity of other signaling such as NFκB for *miR-31* attenuation. Based on these findings, experiments exploring a range of dosages and time points are required to ascertain the specificity of curcumin when abolishing oncogenic signaling, in addition to its effect on AKT that represses C/EBPβ expression. As curcumin is able to down-regulate AKT expression and endogenous *miR-31* expression, its validity as a means of intercepting OSCC could be valuable [41].

Our previous study identified that curcumin activates p38/MAPK, which brings about C/EBPα up-regulation and IGFBP-5 induction; these effects result in tumor suppression in OSCC [31]. This study pinpoints the fact that the EGFR-AKT-C/EBPβ regulatory axis may underlie *miR-31* up-regulation in OSCC. Furthermore, by inactivating AKT, curcumin is able to attenuate both endogenous *miR-31* expression and EGF induced *miR-31* up-regulation. Combining curcumin with an anti-EGFR regimen that targets this specific oncogenic pathway might be a useful strategy for the treatment of OSCC [6].
